# Leadership styles and employee knowledge sharing: Exploring the mediating role of introjected motivation

**DOI:** 10.1371/journal.pone.0257174

**Published:** 2021-09-27

**Authors:** Seemab Chaman, Sehar Zulfiqar, Sadia Shaheen, Sharjeel Saleem

**Affiliations:** 1 Department of Management Sciences, COMSATS University, Islamabad, Pakistan; 2 Department of Management Sciences, National University of Modern Languages, Islamabad, Pakistan; 3 Lyallpur Business School, Government College University, Faisalabad, Faisalabad, Pakistan; Bucharest University of Economic Studies, ROMANIA

## Abstract

Drawing on Social Exchange Theory and Self-Determination Theory, this study examines the impact of three leadership styles (ethical, transformational, and passive avoidant) on employee knowledge sharing. Further, this study explores the mediating effect of introjected motivation in the relationship between three leadership styles and employee knowledge sharing. Using time lag data this study employed a sample of 254 faculty members of public sector universities in Pakistan. Results supported the positive relationship between three styles of leadership and employee knowledge sharing. Moreover, our findings confirmed the mediating role of introjected motivation in the relationship between three leadership styles and employee knowledge sharing. Our study is unique, as it simultaneously examines how various styles of leadership predict introjected motivation and employee knowledge sharing. Implications along with limitations and future research directions are discussed.

## Introduction

Knowledge is a significant intangible organizational asset [1]. With the help of knowledge, an employee becomes more responsive and can deal with unexpected situations [2]. Knowledge sharing is the flow of knowledge among employees that facilitates the creation of new knowledge [3]. In knowledge-intensive organizations, knowledge sharing is the key resource that has immense potential to bring positive outcomes such as creativity [4], firm innovative behavior [5] team performance [6], reduction in production cost [7],and enhancing firm performance including sales growth [8]. Especially in the context of the service industry, knowledge sharing can be a source of competitive advantage The service organization’s performance is highly dependent on how well knowledge is shared between individuals, teams, and organizations. In this case, adopting effective knowledge management strategies allows organizations to deal with complicated issues through collaboration [9]. Though knowledge sharing is paramount and indispensable for every organization but it is difficult to turn individual knowledge into organizational knowledge because employees feel reluctant to share their knowledge [10]. There is considerable research conducted on knowledge sharing in the past few years, but still, there is a lot to be known about the antecedents that foster or restrict knowledge sharing at the workplace [4]. Past research shows the organizations have to reap the culture of knowledge sharing which is largely dependent on the personality of the leader and how the leader motivate employees to engage in knowledge sharing [11]. A perfect leadership style can play an integral role in boosting knowledge sharing behavior in any organization [12]. Knowledge sharing in the organizations can take place in multiple directions such as horizontally (among colleagues at the same level of the hierarchy) and vertically (among the different levels of hierarchy such as knowledge flow from top to bottom or bottom to top). Often the immediate leaders are capable of influencing knowledge sharing within all these directions by developing a conducive or obstructive environment [13]. In this context, leadership styles such as ethical, transformational, and passive avoidant leadership can play a role in enhancing employee’s knowledge-sharing behavior.

However, in modern literature, several organizational and individual factors which have the potential to affect knowledge sharing have been discussed. Such as interpersonal trust [14], organizational culture [15], organizational support [16], and rewards [17], but the role of leader in boosting knowledge-sharing still demand attention by academic researchers [18]. Leader’s behavior has a significant effect on the attitudes and behaviors of their followers [19] or in other words it can be said thatemployees tend to follow their leaders.

Knowledge sharing in the context of universities is an ongoing process. Currently, faculty members are increasingly encouraged to collaborate with other researchers within the department, across departments, across universities domestically and internationally. They are expected to work in teams, learn, collaborate, and share knowledge with other team members to create new knowledge through publications and winning research grants. However, faculty members in the quest of achieving high research performance in a highly competitive environment may not be willing to share their knowledge. In this context, the role of a leader is extremely important because knowledge sharing will be largely dependent on how leaders develop a collaborative working environment based on trust, openness, and ethics. Along with the leadership, it is also important to study the individuals’ psychological needs such as what motivates faculty members to share their knowledge [3]. Thereis a limited investigation done on which leadership style is more appropriate for knowledge sharing [20,21] particularly in public sector universities of Pakistan. Public sector universities are knowledge-intensive and rely on effective knowledge sharing among different departments [22]to develop collaboration and innovation among faculty members [23]. It improves the working of educational institutions by stimulating change and reforms. Therefore,there arises a need for research to know the mechanisms and processes through which leadership influences faculty member’s knowledge sharing.Similarly, the mechanisms between leadership styles and knowledge sharing are still unexplored especially in the context of public sector universities in Pakistan.

Based on the notion of Self-Determination Theory (SDT), it is interesting to know and examine how and why particular human behavior such as knowledge sharing is induced [24] through a particular leadership style. According to SDT, human behavior is shaped by either externally induced motivation, called controlled motivation, or by internally evoked incentives or pressures, called autonomous motivation [25]. We argue that controlled and moderately controlled motivation especially the introjected motivation can significantly impact the knowledge-sharing behavior of employees [26]. In the current study, we propose that an appropriate leadership style can enhance the feeling of worth in employees and build strong relationships which will lead towards increasing their introjected motivation and they will feel obliged to share knowledge [27]. In this study, a deeper understanding of the leadership process is conducted by focusing on different leadership styles and motivations.

Based on the above discussion, the purpose of this study is to examine how various leadership styles such as ethical, transformational, and passive avoidant leadership influence employee knowledge sharing. Further, it investigates the mediating role of introjected motivation in the relationship between leadership styles (e.g., ethical, transformational, and passive avoidant leadership) and employee knowledge sharing.

## Theory and hypotheses

### Ethical leadership and employee knowledge sharing

Knowledge sharing is defined as a set of behaviors that involve an exchange of information or assistance to others’ or ‘it is the act of making knowledge available to others within the organization’ [28]. To influence the interpersonal process of knowledge sharing in organizations, leadership can be seen as an important factor. Knowledge sharing might be predicted by a leader’s ethicality [29,30]. Ethical leadership can be defined as the “demonstration of normatively appropriate conduct through personal action and personal relationship, and the promotion of such conduct to followers through two-way communication, reinforcement and decision making” [31]. The opportunities and motivation provided by ethical leaders to their followers can help to promote knowledge sharing and discourage them from knowledge hiding [32]. In doing so eithical leaders prevent the factors that constrain the mutual sharing of resources among employees, by implementing policies and systems that promote morality [33].

The relationship between ethical leadership and knowledge sharing can be explained through Social Exchange Theory (SET). According to SET, ethical leaders and their followers perceive themselves in a social exchange relationship. When employees receive ethical treatment from their leaders, they feel that their leaders care for them and work with ethics [34]. Due to trust between them the follower’s knowledge sharing and work outcomes will be enhanced. Based on the SET perspective, a leader must be viewed as a striking, realistic, and genuine role model who involve in ethical behavior. Hence,if the leader wants to promote knowledge sharing at the workplace then he/she needs to be an ethical leader [35]. Ethical leadership plays an important role in promoting knowledge-sharing behaviors among employees [36,37] by rewarding those employees who will display pro-social behaviors of knowledge sharing [38]. Based on the above literature, we hypothesize:

*H1*: *Ethical leadership is positively related to employee knowledge sharing*.

### Transformational leadership and employee knowledge sharing

Transformational leadership is defined as a process through which followers are motivated to perform at a higher level by the inspirations provided by their leaders and through a higher level of shared vision [39]. Transformational leaders positively influence employees and enhance their job satisfaction, commitment, and achievement [40]. According to [41] transformational leaders motivate employees to share knowledge in the organization. Transformational leaders have idealized influence, inspirational motivation, intellectual stimulation, and individual consideration [39,42]. According to [43] idealized influence shows the extent to which the individuals engage in behaviors that encourages followers to identify with them. Inspirational motivation explains that an individual puts forth a vision to inspire followers [43]. Intellectual stimulation shows the extent to which individuals challenge existing assumptions and encourage them to take risks. Finally, individual consideration describes the extent to which an individual seeks to meet the individual needs of his or her followers. Therefore, through idealized influence, inspirational motivation, intellectual stimulation, and individual consideration transformational leaders stimulate different ways of thinking among employees, identify new opportunities, identify solutions to problems and enhance the employee’s knowledge-sharing behavior [41]. According to SET, transformational leaders support, stimulate and inspire employees and creates a positive organizational climate, which encourages knowledge sharing, and promotes organizational learning [44]. This positive climate of knowledge sharing with the help of transformational leadership between managers and leaders [45] helps improve employee efficiency and productivity through effective engagement in knowledge sharing across organizations [46]. Therefore, we hypothesize that transformational leadership plays a significant positive role in motivating employees to engage in knowledge sharing in organizations [47].

*H2*: *Transformational leadership is positively related to employee knowledge sharing*.

### Passive avoidant leadership and employee knowledge sharing

Passive avoidant leadership consists of two dimensions: the first dimension is management by exception (passive) which is the active form of behavior and the second dimension is laissez-faire which is considered as a form of non-leadership where individuals avoid leadership by avoiding responsibility and problem solving [48]. Previous research has concluded that passive avoidant leadership is negatively related to organizational commitment, job satisfaction and organizational values.

Limited and unclear evidence is available in the knowledge management literature on the relationship between this style of leadership and knowledge sharing. Previously it was found that the passive avoidant leadership style will not have a positive impact on the knowledge-sharing behavior of employees. The passive avoidant leadership style will not support and facilitate workers to provide and share knowledge [49]. Employees need to have a trusted relationship with their leader so that they can share their problems and also obtain guidance in the time of crises. But when the leader is not available at the time when he/she is needed, it develops a sense of isolation and insecurity among the followers. This sense of isolation and distrust associated with this style of leadership may not promote knowledge-sharing behaviors and will eventually decrease knowledge management activity [50].

The relationship between passive avoidant leadership and knowledge sharing can also be explained through SET [51]. The leader and follower relationship is an exchange relationship with the principle of reciprocity. Followers reciprocate the behavior of their leaders. As discussed earlier, e.g. that the ethical treatment of a leader is reciprocated by the follower through knowledge sharing. In the same way, passive-avoidant leadership (characterized by reactive and non-leadership behaviors) will surely lower knowledge management activity [47] because the followers will not get sufficient knowledge from the leaders due to their passive style of leadership and consequently they will reciprocate through passive behaviors such as limiting their sharing of knowledge with leaders and co-workers. Hence, it is hypothesized that:

*H3*: *Passive avoidant leadership is negatively related to employee knowledge sharing*.

### Introjected motivation as a mediator

According to SDT, human behavior is regulated to the extent to which it is controlled versus autonomous. SDT identifies four types of motivation: external, introjected, identified, and intrinsic motivation [52]. Introjected motivation is the most important form of moderately controlled motivation [53]. In introjected motivation, individuals engage in work activities due to their feeling of obligation but not because they fully internalize the activity itself. It can be argued here that employees with introjected motivation engage in knowledge sharing to improve their self-worth. They want to feel good about themselves and avoid any feeling of guilt and shame to maintain their egos [52]. Controlled motivations that are caused by either external or internal factors such as tangible and intangible rewards will significantly affect people’s behaviors such as employee’s knowledge sharing behavior [54,55]. An anticipated consequence or incentive associated with behavior is related to an individual’s personal and performance-related outcome expectations regarding a given behavior [56]. According to SDT, the benefits or rewards that accumulate for individuals such as soft rewards (relationship with others and reputation) and hard rewards (promotion, financial rewards, and other benefits in the workplace) [57] due to their knowledge-sharing behaviors are the key factors in personal and performance-related outcome expectations [55].

Based on SDT, different types of motivational factors along with different leadership styles help to interpret and explain knowledge-sharing behaviors.Previous studies [9,58] indicate that encouraging individuals to engage in a particular behavior requires motivations associated with an individual’s expectations of getting a favorable outcome. A subordinate’s motivation is affected by a leader’s behavior which in turn influences the outcomes of a task. It shows that how leaders motivate subordinates to involve in knowledge-sharing behaviors.

Ethical leaders may alter the individuals’ behavior by motivating them. Ethical leadership along with introjected motivation is an important tool for encouraging employees to actively share knowledge with coworkers [59]. Employees could be motivated to involve in knowledge sharing due to introjected motivation such as the relationship with a leader, leader’s ability to build an ethical reputation, and leader’s ability to control and administer rewards for desired behaviors [60]. An effective leader focuses on people and creates affiliations with others. He/she will inspire and motivate his/her followers which in turn enhances their knowledge-sharing behavior [61].

The direction provided by ethical leaders to their employees is a key source of guidance at the workplace [31]. Brown et al, (2005) studied ethical leadership from a social learning perspective and found that ethical leaders are role models of appropriate behavior. Unethical leadership and behavior result in an economic downturn and also the cost of such behavior is unbearable [62]. Similarly, according to [51], SET explains that mutual obligations between two parties are created by a give and take relationship [63]. The behavior of both parties affects those exchange relationships [51]. Therefore, we argue that in the context of the moral lens leader’s ethicality will shape the introjected motivation of the employee as the employees will feel obliged to respond to the leader’s ethical treatment through knowledge-sharing behavior [64]. Hence, employee knowledge-sharing behavior can be significantly predicted by ethical leadership through introjected motivation [65].

Similarly, transformational leaders will also affect the introjected motivation for knowledge sharing [9]. To mobilize individuals for achieving organizational goals, the transformational leadership style helps to transform employees’ values and standards to shape their behaviors [46]. Introjected motivation is an important internal regulation and will encourage employees to share knowledge with their colleagues. Introjected motivation helps to promote feelings of worth. The behavior of an individual does not depend on others’ rewards and punishments [66]. Rather, an individual himself monitors and administers the rewards. Introjected motivation helps to motivate employees to maintain and enhance the feeling of worth in their social groups [60]. It is predicted that employee knowledge sharing is positively related to this type of motivation. To maintain feelings of worth in the organization, an employee will be motivated to share knowledge within the organization [25]. Introjected motivation involves individuals’ expectations of getting an implicit reward (personal status and affiliations with others). Introjected motivation involves a high level of personal autonomy, reputation, and relationship [24]. Transformational leadership is linked with different motivational outcomes in employees including empowerment, autonomous motivation, and controlled motivation. Leaders can enhance knowledge-sharing behavior among employees by fostering introjected motivation [53,58,67] and in this case, transformational leaders can foster introjected motivation through change, inspiration, and empowerment. Consequently, introjected motivation can serve as an important regulation for empowered employees to actively share knowledge with co-workers. Similarly, the employee will solve tasks according to managers and colleagues and be involved in knowledge-sharing behaviors if he wants to fit in and gain acceptance in the organization [68].

**Fig 1:**
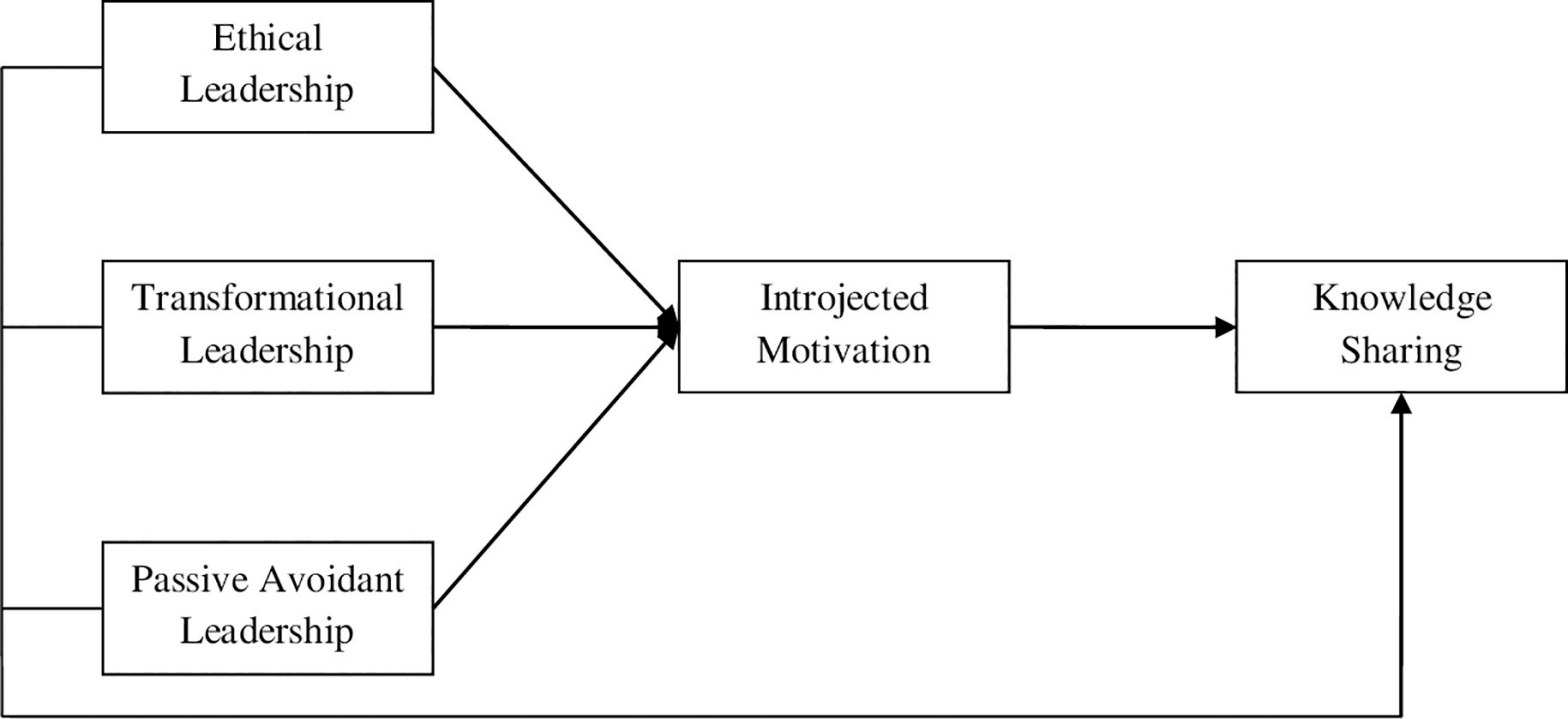
Proposed theoretical framework.

Passive avoidant leadership is based on punishments and avoidance and it strives to maintain the status quo through postponement, nonattendance, and unresponsiveness. When the leaders avoid responsibility and are unresponsive, subordinates feel a lack of psychosocial support, and mentoring [69]. Passive avoidant leaders may leave their subordinates unattended and cultivate careless and passive behavior among their subordinates [49]. Therefore, we argue that the passive avoidant style of leadership will negatively affect the introjected motivation of employees that will eventually lower their knowledge sharing Hence, it is hypothesized that:

*H4a*: *Introjected motivation mediates the relationship between ethical leadership and employee knowledge sharing*.
*H4b*: *Introjected motivation mediates the relationship between transformational leadership and employee knowledge sharing*.
*H4c*: *Introjected motivation mediates the relationship between passive avoidant leadership and employee knowledge sharing*.
*Based on the above discussion Fig 1 shows the proposed theoretical framework of the current study:*

### Methodology

Data for the current study were collected from faculty members working as full-time employees in several public sector universities situated in four major cities of Pakistan namely Lahore, Faisalabad, Rawalpindi, and Islamabad. Data were collected from different departments such as management sciences, history, social sciences, geography, computer sciences, and engineering.

We collected the data only from the teaching staff such as lecturers, assistant professors, and professors. All the employees were contacted through their deans and directors. We used all the research protocols for data collection. Initially, we took permission from the heads of the department and convey to them the purpose of data collection. Secondly, before data collection, it was elaborated to the faculty members that their responses will be used only for the completion of this research work. Their responses will be kept confidential and will be used in an aggregate manner. There are no right and wrong answers we just need your valuable opinion. All the faculty members have full freedom to participate or not participate in this study. Additionally, at any stage of data collection, all the participants are free to quit from this study without any penalty. In this way, written informed consent was taken from the respondents.

To avoid the possible threat of common method bias, data were collected in three-time lags. In time lag 1 data were collected on three types of leadership styles such as ethical leadership, transformational leadership, and passive avoidant leadership. In time lag 2, (after one month) data were collected regarding introjected motivation. In time lag 3 (again after a one-month time interval), data were collected on knowledge sharing.

A total of 500 respondents were approached. In time lag 1 (T1), 500 questionnaires were distributed. We received 440 filled questionnaires. Among 440 there were 25 questionnaires were discarded due to having incomplete information. In time lag 2 (T2), only those respondents were contacted who filled the questionnaire at time lag 1 (T1). At this time total of 415 questionnaires were distributed and 387 were returned but 35 questionnaires were discarded due to having incomplete information. At time lag 3 (T3), 352 questionnaires were distributed and 309 were returned. Among 309 questionnaires, 55 questionnaires were discarded due to having missing information. We used a total of 254 questionnaires for data collection.

According to demographics results, there were 43.0% females and 57.0% male; 18.9% belong to the age group of 21–25 years, 36.2% belong to the age group of 26–30 years, 22.4% belong to the age group of 31–35 years, 13.0% belong to the age group of 36–40 years and 9.5% belong to the age group of above 40 years. According to educational status, 9.8% had a Master’s degree, 57.9% had MS/Mphill degree and 32.3% had a Ph.D. degree. Related to employee tenure 42.9% has 1–3 years of experience, 22.8% had 4–6 years of experience, 14.6% had 7–9 years of experience 9.1% had 10–12 years experience and 10.6% has above 12 years experience. Demographics information of all the respondents has been presented in Table 1.

**Table 1:** Profile of respondents.

Demographics Characteristics	Percentage (%)
**Gender**	
Male	57.5%
Female	42.5%
**Age**	
21–25 Years	18.9%
26–30 Years	36.2%
31–35 Years	22.4%
36–40 Years	13.0%
Above 40 Years	9.4%
**Education**	
Masters	9.8%
Ms/MPhil	57.9%
PhD	32.3%
**Tenure**	
1–3 Years	42.9%
4–6 Years	22.8%
7–9 Years	14.6%
10–12 Years	9.1%
Above 12 Years	10.6%

### Measures

The measures for all variables were adapted from the pervious research work. The scales adapted for the current study were widely used to measure the variables in the various organizational settings including universities. The language of the questionnaire was English and the wording of the items was adapted according to the universities context. Previously, these scales were also adapted by the researchers according to their study settings [70]. All items were rated on a five-point Likert scale (1 = strongly disagree, 2 = disagree, 3 = neutral, 4 = agree, 5 = strongly agree).

### Ethical leadership

Ten items scale of ethical leadership (EL) developed by [31] was used in this research.Previously this scale is widely used by researchers to measure the ethical leadership in various organizational settings such as insurance companies [71], banking [72], and also it is employed by studies conducted in the context of universities [73–76]. The sample items include “My supervisor listens to what employees have to say”, “My supervisor disciplines employees who violate ethical standards”. The Cronbach’s Alpha for this measure in the present study was **(0.87).**

### Transformational leadership

Eight item scale of Transformational leadership (TFL) developed by [77] was used in this research. This scale is used in the previous studies to measure transformational leadership in various industries [78] including universities [79,80]. The sample items include ‘the supervisor can understand my situation and gives me encouragement and assistance’; ‘The supervisor encourages me to take challenges’. The Cronbach’s Alpha for this measure in the present study was **(0.84).**

### Passive avoidant leadership

Passive avoidant leadership (PAL) was measured using the eight-item scale developed by [81]. This scale is adapted by researchers to measure the passive avoidant leadership style in organizations such as information technology firms [82], and universities [83,84]. The sample items include ‘My supervisor reacts to problems, if serious’, ‘My supervisor reacts to failure’. The Cronbach’s Alpha for this measure in the present study was **(0.75).**

### Introjected motivation

Introjected motivation was measured using a four-item scale developed by [85]. The sample items include ‘My knowledge sharing would expand the scope of my associations with other members in my company’ and ‘my knowledge-sharing would strengthen the tie between the existing members in my company and myself.’The Cronbach’s Alpha for this measure in the present study was **(0.78).**

### Knowledge sharing

An 8-item scale of knowledge sharing developed by [86] was used in this research. This scale is employed by researchers to measure knowledge sharing in manufacturing and non- manufacturing firms [87], high-technology companies [88] and educational context [16]. A sample item is “In daily work, I take the initiative to share my work-related knowledge with my colleagues”. The Cronbach’s Alpha for this measure in the present study was **(0.84).**

### Data analysis

Data analysis was carried out by using SPSS 20. Several tests have been carried out to examine the data such as reliability analysis, correlation analysis, regression analysis, and mediation analysis. Regression and mediation analysis were carried out by using PROCESS macro by Hayes [89]. The absence of common method bias was also ensured with the help of Harman’s one-factor test. According to the results of Harman’s one-factor test the total variance explained by one factor was 28.58%. Which was far less than 50%. Therefore, we claim the absence of common method bias in the present study. Moreover, data collection on different time lags also helped in eliminating the issue of common method bias and social desirability.

### Correlation analysis

Before hypotheses testing Pearson correlation analysis was performed to check the association between all the theoretical variables. According to the correlation analysis of the study ethical leadership positively and significantly related to transformational leadership (*r* = 0.62, *P <*0.5) passive avoidant leadership (*r* = 0.29, *P <*0.05) interjected motivation (*r* = -0.64, *P <*0.05) and knowledge sharing (*r* = -0.52, *P <*0.05). Transformational leadership positively and significantly related to passive avoidant leadership (*r* = 0.13, *P <*0.01) introjected motivation (*r* = -0.62, *P <*0.05) and knowledge sharing (*r* = -0.53, *P <*0.05). Intorjected motivation positively and significantly related to knowledge sharing (*r* = o.47, *P <*0.05). (See Table 2).

**Table 2:** Correlation analysis.

		1	2	3	4	5
**1**	Ethical Leadership	**(0.87)**				
**2**	Transformational leadership	0.62**	**(0.84)**			
**3**	Passive Avoidant leadership	0.29**	0.13*	**(0.75)**		
**4**	Introjected Motivation	0.64**	0.62**	0.39**	**(0.78)**	
**5**	Knowledge Sharing	0.52**	0.53**	0.49**	0.47**	**(0.84)**

*p < .10

**p < .05, ***p < .01.

### Test of hypothesis (1–3)

We analyzed the hypotheses by utilizing the method suggested by [90]. The hypotheses were tested by utilizing PROCESS macro by [89]. We used model 4 to test the mediating role of introjected motivation between all independent variables such as ethical leadership, transformational leadership, and passive avoidant leadership and knowledge sharing.

The results of hypotheses 1–3 have been reported in Table 3. Hypothesis 1 stated ethical leadership was positively related to employee knowledge sharing. The results of the study show support for hypothesis 1 (*b* = *-*0.35, *p <*0.01); therefore, hypothesis 1 accepted. Hypothesis 2 stated, transformational leadership was positively related to employee knowledge sharing which was also supported by statistical results (*b* = *-*0.38, *p <*0.01); thus hypothesis 2 was accepted. Hypothesis 3 stated passive avoidant leadership is negatively related to employee knowledge sharing which was not supported by statistical results (*b* = 0.36, *p <*0.01). Thus, we found a positive relationship between passive avoidant relationshiop and knowledge sharing. Therefore, hypothesis 3was rejected.

**Table 3:** Regression analysis.

Direction of relationships	Path Coefficients	SE	P-Values
Ethical leadership Knowledge Sharing	0.35***	0.64	0.000
Transformational leadership Knowledge Sharing	0.38***	0.06	0.000
Passive avoidant leadership Knowledge Sharing	0.36***	0.05	0.000

*p < .10, **p < .05

***p < .01.

### Test of hypothesis (4–6)

#### Mediation analysis

According to hypothesis 4a ethical leadership was expected to have an indirect effect on employee knowledge sharing through introjected motivation. The indirect effect of ethical leadership on employee knowledge sharing was proved as depicted by a 95% confidence interval which did not include zero [0.036; 0.262]. Based on statistical results a mediation in the direct relationship between ethical leadership and employee knowledge sharing was proved. Thus hypothesis 4 was accepted. Hypothesis 4b stated transformational leadership was expected to have an indirect effect on employee knowledge sharing through introjected motivation. The indirect effect of transformational leadership on employee knowledge sharing was proved as depicted by a 95% confidence interval which did not include zero [0.035; 0.242]. Based on statistical results a mediation in the direct relationship between transformational leadership and employee knowledge sharing was proved.

According to the hypothesis 4c passive avoidant leadership was expected to have an indirect effect on employee knowledge sharing through introjected motivation. The indirect effect of passive avoidant leadership on employee knowledge sharing was proved as depicted by a 95% confidence interval which did not include zero [0.053; 0.237]. Based on statistical results a mediation in the direct relationship between passive avoidant leadership and employee knowledge sharing was proved. Thus relying upon statistical evidence hypotheses 4a, ab and 4cwereaccepted. (See Tables 4–6).

**Table 4:** Results of the mediation analyses of EL, IM, and KS (without covariates).

	Coefficient	SE	Bootstrap 95% CI
***IV to mediator (A path)***			
**EL→ IM**	0.613***	.0461	
***Mediator to DV (B path)***			
**IM→KS**	0.231***	0.067	
***Total effect of IV on DV* (*C path*)**	0.498***	.050	
***Direct effect of IV on DV* (*C path*)**	0.356***	.064	
***Indirect effect of IV on DV through proposed mediator***			
**EL→ IM→ KS**	0.141***	.057	[0.036; 0.262]

Note, EL = Ethical Leadership, IM = Introjected Motivation, KS = Knowledge Sharing.

*p < .10, **p < .05

***p < .01.

**Table 5:** Results of the mediation analyses of TFL, IM, and KS (without covariates).

	Coefficient	SE	Bootstrap 95% CI
***IV to mediator (A path)***			
**TFL → IM**	0.608***	.047	
***Mediator to DV (B path)***			
**IM →KS**	0.224***	0.066	
***Total effect of IV on DV (C path)***	0.518***	.051	
***Direct effect of IV on DV (C path)***	0.382***	.064	
***Indirect effect of IV on DV through proposed mediator***			
**TFL → IM→ KS**	0.142***	.051	[0.035; 0.242]

Note: TFL = Transformational Leadership, IM = Introjected Motivation, KS = Knowledge Sharing.

*p < .10, **p < .05

***p < .01.

**Table 6:** Results of the mediation analyses of PAL, IM, and KS (without covariates).

	Coefficient	SE	Bootstrap 95% CI
***IV to mediator (A path)***			
**TFL → IM**	0.404***	.059	
***Mediator to DV (B path)***			
**IM →KS**	0.330***	0.055	
***Total effect of IV on DV (C path)***	0.502***	.056	
***Direct effect of IV on DV (C path)***	0.368***	.057	
***Indirect effect of IV on DV through proposed mediator***			
**TFL → IM→ KS**	0.133***	.047	[0.053; 0.237]

Note: PAL = Passive Avoidant Leadership, IM = Introjected Motivation, KS = Knowledge Sharing.

*p < .10, **p < .05

***p < .01.

## Discussion

In sum, we found good support for the proposed model. Results of the study, show that ethical, and transformational, and passive avoidant leadership has a significant positive effect on employee knowledge sharing. We also found good support for mediating relationships. According to results, ethical, and transformational leadership have an indirect effect on employee knowledge sharing through introjected motivation. Findings are supported by SET, which implies that positive behaviors are reciprocated with positive acts. When employees are fairly treated by their leaders they feel obliged and trusted; therefore they are more motivated to respond to the ethical treatment of the leader by engaging in prosocial behaviors such as citizenship behavior, organizational commitment, and knowledge sharing. Ethical treatment from the leader has a positive impact on the personal and professional lives of the followers. Therefore, in line with their leader’s fair actions, the followers also try to practice ethical and balanced practices such as not to deceive coworkers, participate in the goodwill, growth, and better functioning of the organizations through knowledge sharing when needed.

Similarly, our findings suggest that transformational leaders motivate their followers to become more engaged in their work and exhibit extra-role behavior such as knowledge sharing behavior. Our findings are consistent with the previous research [46,91]. We argue that in transformational leadership, a leader emphasizes and inspires followers in achieving organizational goals which can only be possible when employees are willing to exchange their knowledge. Especially the tacit knowledge that employees hold is fundamental in achieving organizational goals. Transformational leaders can evoke the significance of the outcome of any task in the followers’ mind, encourage them to participate in the accomplishment of organizational goals by going beyond the formal requirements and personal interests. This sense of organizational responsibility educes followers to be more motivated to share their knowledge without fear and hesitation with their colleagues for the smooth achievement of organizational goals.

However, we found that passive avoidant leadership was positively related to knowledge sharing behavior. One possible reason for this finding is that the passive avoidant leaders give autonomy to their subordinates for the completion of tasks in some situations as leaders themselves avoid taking charge and responsibility. Previous literature shows that autonomy motivates employees and encourages interactions among co-workers to search for information and create new knowledge [92]. Therefore, it can be argued that the passive avoidant leadership style leads to autonomy which eventually increases the knowledge management activity of the employee, especially at the personal level.

The notion of SDT [67], helps us in justifying the mediating role of introjected motivation to promote knowledge-sharing behavior. Introjected motivation motivates employees to foster positive behaviors through soft rewards such as (reputation and relationships with others). According to SET employees under ethical and transformational leadership have introjected motivation and display positive behaviors such as knowledge sharing due to the principle of reciprocity. The positive impact of introjected motivation on employee knowledge sharing is consistent with the previous studies as well [53,93]. All three leadership styles (ethical, transformational, passive avoidant) promotes autonomy-oriented motivation by enhancing individual’s perceptions of autonomy which encourages them to share their knowledge. Authority comes with responsibility, therefore employees feel obliged to share their knowledge for the successful completion of their duties. Moreover, introjected motivation helps employee’s to internationalization of organizational goals which is very important in motivating employee’s knowledge-sharing behaviors as they perceive organizational goals as their own goals.

### Theoretical implications

The current study significantly contributes to the knowledge management and leadership literature in the following ways;

First, to the best of our knowledge, this is the first study to explore the impact of several types of leadership such as ethical, transformational, and passive avoidant on employee knowledge sharing behavior in public sector universities of Pakistan. By doing so, we tried to explore the importance of each leadership style in enhancing employee knowledge sharing. Which helped us in identifying which leadership styles promote knowledge sharing and how to strengthen that specific leadership style.

Secondly, our study makes a unique contribution in the field of knowledge management by exploring the mediating role of introjected motivation in the leadership styles and knowledge sharing relationship. Therefore our study by using SET and SDT highlights how various leadership styles lead to introjected motivation which eventually fosters the knowledge-sharing behavior, which has been rarely investigated.

### Practical implications

The current study provides a valuable understanding of how organizations can promote employee knowledge-sharing behaviors through apporiate leadership style. Our findings imply that ethical and transformational leaders are the key ingredients for knowledge sharing in the public sector universities of Pakistan and introjected motivation fuels this fire.

Our findings suggest that ethical leadership promotes employee knowledge-sharing behavior in public sector universities. A leader should decide rewards and punishment in a justified manner. A fair and equitable treatment evokes a sense of ethical responsibility among the followers regarding the organization. Therefore, universities need to develop a culture of ethical leadership through different ways such as pieces of training, workshops, and role modeling. The morality, integrity, and ethical treatment of ethical leaders might motivate followers to share their knowledge with the other organizational members for the shared benefit of the organization. Thus, universities should encourage and train leaders to practice ethical leadership. Faculty and staff follow the footsteps of their leaders. Therefore, leaders should ethically share their knowledge and reward subordinate knowledge sharing fairly through monetary and non-monetary rewards, it will develop a sense of security among subordinates that their knowledge sharing will not lead to any kind of exploitation.

Secondly, management should encourage a transformational style of leadership in the universities and involve faculty in decision making and other important activities such as allocation of courses, management of timetables, and student selection for research supervision. All these factors nurture a sense of organizational commitment, involvement, and self-determination to serve the university at the optimal level. Consequently, faculty will feel a sense of personal concern and responsibility in achieving university goals which in turn force them to share their knowledge to boost the university’s performance. Leadership at the departmental level should foster knowledge sharing by developing collaborative work culture and create opportunities for common projects.

Thirdly, in universities, passive avoidant leadership needed to be addressed properly. Management of the public sector universities should discourage passive avoidant leadership styles and encourage leaders to take active actions in guiding and liaising with their followers. There should be behavior modification training for the leaders. Management should foster and demonstrate the advantages of ethical and transformational leadership. So that non-leaders can alter their leadership style. Lastly, leaders should motivate their faculty members through soft rewards such as allocation of titles, appreciation, and encouragement on achieving organizational goals.

### Limitations and future research directions

This study makes a unique contribution by empirically and theoretically examining the role of various leadership styles on employee knowledge sharing behavior in the context of Pakistani public sector universities. However, this study has some limitations as well. One limitation was time and resource constraints in data collection due to which data was collected from few universities of one province in Pakistan Therefore; the sample was not representative of all universititess. Future research can be carried out by collecting data both from public and private sector universities. Additionally, a comprehensive picture can be drawn by the comparison of leadership styles between public and private sector universities. Secondly, the measures were self-reported; the issue of common method bias may arise. Although we employed *Harman’s single factor test* to identify the problem of common method variance it is exploratory thereforefuture studies may address this issue through data collection from multiple sources and using more robust statistical techniques. Thirdly we only tested the role of leadership styles on employee knowledge sharing behavior through the mediating role of one factor i.e. introjected motivation. There might be other factors that can play the role of a moderator such as personality types, career orientation, and organizational culture. We suggest exploring the role of moderators in future studies Lastly, we collected the data on knowledge sharing through a self-reported measure, however, there are some forms of knowledge sharing in universities, e.g. co-authorship, and co-teaching that can be used as proxies to measure knowledge sharing of faculty. Future studies may consider e.g. using number of papers co-authored with colleagues as a proxy for knowledge sharing in the universities instead of measuring it through questionnaires.

## Supporting information

S1 Data (ZIP)
